# Development of Cellulose Acetate Spherical Microparticles by Means of Melt Extrusion of Incompatible Polymer Blend

**DOI:** 10.3390/polym17152118

**Published:** 2025-07-31

**Authors:** Masaya Omura, Keiko Kobayashi, Kanji Nagai, Shu Shimamoto

**Affiliations:** 1 Business Strategy, Healthcare SBU, Daicel Corporation, Minato-ku, Tokyo 108-0075, Japan; 2 Graduate School of Natural Science and Technology, Kanazawa University, Kanazawa-Shi 920-1192, Japan; 3 CAFBLO Business Strategy, Material SBU, Daicel Corporation, Himeji-Shi 671-1283, Japan; 4 Life Sciences R&D Center, PharmaTek BU, Life Sciences SBU, Daicel Corporation, Myoko-Shi 944-8550, Japan; 5 Business Development Center, Innovation and Business Development Headquarters, Daicel Corporation, Minato-ku, Tokyo 108-0075, Japan

**Keywords:** cellulose acetate, incompatible polymer, microparticles, melt blending, plasticizer migration

## Abstract

Cellulose acetate (CA), commercially produced from natural cellulose, is one of the promising candidates to solve the microplastic issue. In this study, attempts were made to prepare CA microparticles by means of melt extrusion of incompatible polymer blends comprising CA with plasticizer (triacetin (TA)) and polyvinyl alcohol (PVA) followed by selective removable of TA and PVA. As implied by semi-theoretical equation previously established by Wu (Wu’s equation), particle size decreased with increasing shear rate or decreasing viscosity ratio of polymers. CA microparticles with a controlled size of 2–8 μm, narrow particle size distribution, and smooth surface were successfully obtained. Efforts were made to determine the numerical solution of Wu’s equation to compare them with observed particle size. To this end, interfacial tension between dispersed and matrix phases to be incorporated in the equation was determined by group contribution methods. The root mean squared error (RMSE) between the observed and calculated particle size was unsatisfactorily large, 4.46 μm. It was found that one of the possible reasons for the limited prediction accuracy was migration of TA from the dispersed to matrix phase affecting the viscosity ratio. Further efforts will be required to achieve a better prediction.

## 1. Introduction

Polymer microparticles for cosmetics and other personal care applications have been generally made of plastics produced from fossil resources. Common polymers for microparticles are polyamides, silicone, and acrylic polymers [1,2,3]. Microparticles improve or enhance the texture of cosmetic products. The requirements of microparticles for the optimal sensory appeal are a spherical shape, smooth surface, and diameter within 1 to 15 μm [4].

However, aspects in relation to the harmful environmental effect have limited the advancement in the use of these polymers [5,6]. Utilization of biodegradable polymers in lieu of conventional polymers for microplastics is a possible solution to the issue.

The general platform for the preparation of spherical microparticles is a polymer phase separation system comprising the dispersed and matrix phases, where the matrix is eventually removed leaving the dispersed phase as microparticles. General strategies for preparation of spherical polymer particles by means of polymer phase separation system are enumerated in Table 1. Either (A) monomer–solvent, (B1) compatible polymer–solvent, (B2) incompatible polymer–solvent, or (C) incompatible polymer–polymer systems can be employed as precursor of microparticles.

Polymer spherical particles can be prepared by polymerization of monomers in a non-solvent of the resultant polymer followed by removal of non-solvent (Strategy “A” of Table 1). Spherical polymethyl methacrylate particles have been commercially produced by this method; it is considered the current major texture enhancer used in cosmetics industries. When water is used as a non-solvent, this method is considered cost-competitive. However, few biodegradable polymers are suitable for this method, as their molecular weights are generally too high to facilitate this strategy. Consequently, Strategy “A” is not effective for the preparation of microparticles from cellulose acetate (CA). Polymer–solvent systems, irrespective of their compatibility, have potential as precursors for preparation of spherical polymer particles, as shown in Strategies “B1” and “B2” of Table 1. Unfortunately, these methods usually involve volume shrinkage of the dispersed phase during preparation, which is due to evaporation of the non-solvent from the dispersed phase, losing sphericity and surface smoothness. For these reasons, Strategies “B1” and “B2” were not pursued in this study. In our view, for cellulose acetate, the most promising preparation route in terms of shape of microparticles is emulsification of incompatible polymer–polymer system followed by quenching and removal of the matrix polymer (Strategy “C” of Table 1). In this method, unlike what are commonly referred to as melt blending or polymer alloying for improvement of their physical properties, emulsification is carried out to obtain spherical microparticles. It was reported that the polyamide 12/polyethylene glycol (PEG) system leads to spherical particles of polyamide 12, which are unfortunately not biodegradable, by removing the PEG matrix with water [7]. Likewise, spherical microparticles of cellulose acetate butyrate (CAB) were prepared by the same principle [8]. The product has not been commercially produced to the best of the authors’ knowledge. The potential issues for CAB microparticles are odor due to trace amounts of residual (or released) butyric acid and insufficient biodegradability; there has been no publication yet offering sufficient evidence on biodegradability of CAB. It was reported that CAB even inhibits biodegradability of polybutylene succinate (PBS) when incorporated to PBS at 10% [9].

The reaction of naturally occurring cellulose with acetic anhydride leads to CA, which has been commercially produced for decades for eye-wear flame and other applications, and proven biodegradable [10]. In this study, attempts were made to prepare CA spherical microparticles with a diameter of 1 to 15 μm. We selected the method of emulsification of incompatible polymer–polymer (Strategy “C”)), CA and triacetin (TA) mixture as one polymer, and polyvinyl alcohol (PVA) as another one.

**Table 1:** Strategies for the preparation of spherical polymer particles.

Strategy ID	Method		Advantage	Disadvantage	Example	Ref.
	To Employ	To Utilize				
A	Monomer–solvent system	Polymerization for phase separation. Matrix (non-solvent) to be removed.	Water or ordinary solvent can be utilized as the non-solvent; less waste solvents.	Biodegradable polymer options suitable for the principle are few.	Polymethyl Methacrylate	[11,12]
B1	Compatible polymer–solvent system	Quenching below UCST for phase separation. Matrix (non-solvent) to be removed.	High productivity and less waste solvents.	Spherical particles with smooth surfaces are generally difficult to obtain.	Polybutylene Succinate	[13]
B2	Incompatible polymer–solvent system	Emulsification followed by quenching to fix phase separation morphology. Matrix (non-solvent) to be removed.	Any solvent–soluble polymers assume potential as feedstock.	ibid.	Cellulose acetate	[14,15]
C	Incompatible polymer–polymer system	Emulsification followed by quenching to fix dispersed/matrix morphology. Matrix (polymer) to be removed.	Spherical particles with smooth surfaces are relatively easy to obtain.	Polymers meeting criteria of solubility/insolubility and melting behavior are limited. Small particle size at high volume fraction (hence high productivity) requires rigorous design of process parameters.	Polyamide 12 Cellulose Acetate Butylate	[7,8]

It was reported that the morphology of the dispersed phase depends on the volume fraction of the dispersed phase polymer and the corresponding percolation threshold. This occurred at lower volume fractions in systems with smaller dispersed phase sizes [16,17]. The target particle diameter within 1–15 μm is so small that rigorous design of melt extrusion including selection of polymers are inevitable in the authors’ view. From this perspective, on one hand, a limited volume fraction of dispersed phase polymer is preferable. On the other hand, a limited volume fraction of dispersed phase leads to problems of limited productivity of polymer particles and wastes from matrix polymer. Even if the waste could be reduced by recycling the matrix polymer, the volume fraction of the dispersed phase should be increased as much as possible for the sake of the economy. To this end, rigorous design of recipes for melt blending components and blending conditions are indispensable to obtain microparticle with the target diameter at a high volume fraction for the dispersed phase.

According to the work of Wu [18], parameters other than the volume fraction governing the diameter of dispersed phase are the interfacial tension and the viscosities of the two polymers, and the shear rate of blending as shown in Equation (1).

(1) $$Rn=4\left(\frac{\gamma_{ij}}{{V\eta }_{m}}\right){\left(\frac{\eta_{d}}{\eta_{m}}\right)}^{\pm 0.84}$$

where *Rn* is the average particle size, γ_ij_ is the interfacial tension, V is the shear rate, η_m_ is the viscosity of the matrix, and η_d_ is the viscosity of the dispersed phase. The exponent 0.84 is positive for a viscosity ratio (η_d_/η_m_) greater than 1 or negative for a ratio less than 1 [19].

The primary objective of this study is to prepare CA microparticles with diameters ranging from 1 to 15 μm by melt extrusion and selective removal of the matrix phase, without compromising the volume fraction of the dispersed phase. Wu’s equation was employed as a guideline for particle preparation.

## 2. Materials and Methods

### 2.1. Materials

CA with a degree of acetyl substitution of 2.45 (L-40, Daicel Corporation, Osaka, Japan), PVA with a high molar weight (PVA_H_, G-polymer BVE8049P, Mitsubishi Chemical Group Corporation, Chiyodaku, Tokyo, Japan), and PVA with a low molecular weight (PVA_L_, G-polymer AVE8077P, Mitsubishi Chemical Group Corporation, Chiyodaku, Tokyo, Japan) were dried at 80 °C for 20 h before use. The moisture contents of CA, PVA_H_, and PVA_L_ were 0.6 wt%, 0.9 wt%, and 0.7 wt%, respectively. Reagent grade TA (Sigma-Aldrich, St. Louis, MO, USA) was used without further purification. The materials used in this study are listed in Table 2.

Molecular weights of PVA_H_ and PVA_L_ were measured by means of gel permeation chromatography (GPC, Shimadzu LC-20, Kyoto, Japon) using 0.2 M NaNO_3_/Methanol = 7/3 solution and TSKgel α-M column (TOSO Co., Ltd., Chuo-ku, Tokyo, Japan) calibrated with PEO/OEG standards.

Molecular weights of CA were measured by means of GPC using acetone as eluent and TSKgel GMPWXL column (TOSO Co., Ltd., Chuo-ku, Tokyo, Japan) calibrated with polymethyl methacrylate standards.

### 2.2. Preparation of Cellulose Acetate Spherical Particles

Process 11 parallel twin-screw extruder (Thermo Fisher Scientific Inc., Waltham, MA, USA) with an 11 mm shaft diameter, a length-to-diameter (L/D) ratio of 40, a 0.25 mm tip clearance, and the standard shaft configuration setup was employed for melt blending. CA power and TA liquid (either CA/TA 83/17 wt% or 80/20 wt%) were fed to the extruder to melt blending at 230 °C with a rotation speed of 80 rpm. The resultant CA/TA pellets and PVA (either PVA_H_ or PVA_L_) pellets were fed to the extruder for melt blending at 230 °C with a rotation speed within 40–160 rpm to obtain CA/TA/PVA pellets, where the rotation speed was chosen considering shear rate design.

The blending temperature of 230 °C was determined because of the following preliminary observations: a blending at a temperature well below 230 °C tended to result in an insufficient blend of a CA/TA/PVA ternary system; a blending at a temperature well over 230 °C tended to result in colored pellets presumably because of thermal decomposition of CA.

**Figure 1:**
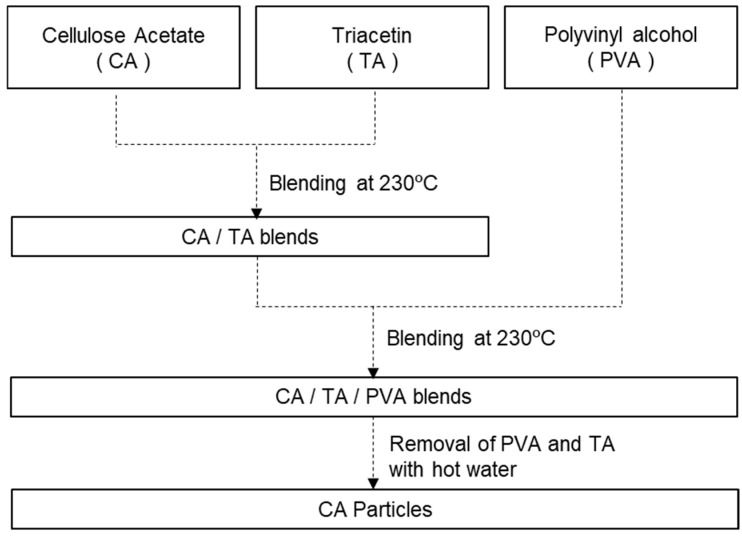
Schematic representation for preparation of CA particles by means of melt blending of CA/TA/PVA ternary system. A twin-screw extruder with a screw diameter of 11 mmφ and L/D of 40 was used for the blending.

The resulting pellets were washed with hot water at 80 °C at a weight ratio of pellets/water of 1/10 and a stirring revolution of 300 rpm for 30 min to remove PVA and TA from the CA/TA/PVA ternary system. The hot water wash was repeated four times in total to obtain wet powders. The wet powders were dried at 80 °C for 20 h. The relevant experimental procedure is illustrated in Figure 1. The dried powders were pulverized by a milling device (Dryburst DB-100S, Sugino Machine Co., Namerikawa, Toyama, Japan) to obtain CA microparticles.

The melt viscosities at the blending temperature (230 °C) of CA/TA binary system, neat PVAs, and PVA/TA binary systems were measured as a function of shear rate by means of a dynamic viscoelasticity measurement device (Capillary Rheometer Capilograph Model 1B, Toyo Seiki Seisaku-sho, Ltd., Tokyo, Japan).

Shear rate of melt blending was calculated by Equation (2) following Wagner JR [20].

(2) $$V=\pi D\frac{N}{h}$$

where V is the shear rate, D is the outer diameter of the rotor, N is the rotor speed, and h is the tip clearance. The blending conditions for the ternary mixture of CA/TA/PVA and the viscosity of the dispersed phase and matrix under the corresponding blending conditions were determined in Supplementally Materials shown in Figure S1.

### 2.3. Calculation of Particle Size by Means of Semi-Theoretical Equation Established by Wu (1987) [18]

Equation (1) has been demonstrated to be valuable in numerous studies following Wu’s work [19,21,22]. The parameters that constitute Equation (1) are the interfacial tension γ_12_, shear rate V, and the viscosities of the dispersed phase η_d_ and the matrix phase η_m_, respectively. Therefore, the equation should hold even if either or both dispersed phase and matrix phase are composed of multiple components. The parameters of Equation (1) are described below.

#### 2.3.1. Surface Tension Components

Surface tension γ of material at 25 °C was determined by the parachor group contribution method established by Sugden and Quayle [23,24]; γ at 230 °C was derived from γ at 25 °C by means of temperature dependence of molar volume [25]. γ at 230 °C was divided into surface tension components γ^d^ (dispersive component) and γ^p^ (polar component) by means of solubility parameter components γ^d^ and γ^p^ at 230 °C following Lee [26]. γ^d^ and γ^p^ at 230 °C were determined by the group contribution method following van Krevelen and Hoftyzer [27,28]. The flowchart for calculation of γ^d^ and γ^p^ is shown as Supplementary Material of this paper (Tables S1–S4, Figure S2).

#### 2.3.2. Interfacial Tension

Interfacial tension between materials i and j (γ_ij_) was calculated by Equation (3) following Wu [29,30].

(3) $$\gamma_{ij}=\gamma_{i}+\gamma_{j}-\frac{4\gamma_{i}^{d}\gamma_{j}^{d}}{\gamma_{i}^{d}-\gamma_{j}^{d}}-\frac{4\gamma_{i}^{p}\gamma_{j}^{p}}{\gamma_{i}^{p}-\gamma_{j}^{p}}$$

#### 2.3.3. Spreading Coefficient

The spreading coefficient of dispersed component i on dispersed component j in matrix k of i/j/k ternary system (λ_ij_) was obtained by Equation (4) following Hobbs [31].

(4) $$\lambda_{ij}=\gamma_{jk}-\gamma_{ik}-\gamma_{ij}$$

### 2.4. Characterization of CA Microparticles

#### 2.4.1. Residual Amount of TA and PVA in Spherical Microparticles

The residual amounts of TA and PVA in CA microparticles were determined by means of HPLC (high performance liquid chromatography, LC-10AD, Kyoto, Japan) with an eluent of 0.1 M NaCl at a flowrate of 0.5 mL/min by an injection of 50 μL of extract from CA microparticles. To prepare the extract for measurement, 2 g of CA microparticles and 10 g of water were placed in a vial, stirred for 1 h, and left to stand overnight. It was subsequently centrifuged at 5000 rpm for 10 min to obtain the extract as supernatant.

#### 2.4.2. Average Particle Size and Its Distribution

Particle size distribution based on number of CA microparticles was measured by a laser diffraction particle size analyzer (Laser Particle Size Analyzer LA-960, Horiba, Ltd., Kyoto, Japan). Average particle size was expressed on a number basis.

#### 2.4.3. Determination of Melting Temperature and Glass Transition Temperature for CA, PVA and Blends

Differential scanning calorimetry (DSC) was performed on Differential Scanning Calorimeter DSC7000X, Hitachi High-Tech Solutions Corporation, Tokyo, Japan. For the DCS measurement, CA in power form was used as received; other materials were pulverized with liquid nitrogen before the measurement. CA, PVA, and relevant blends (CA/TA, PVA/TA, and CA/TA/PVA) were placed in aluminum DSC pans. Sample weight was between 2 and 6 mg. Samples were heated from −20 °C to 250 °C at 10 °C/min for a total of two scans. The cooling rate was 10 °C/min between the first and second scans. Melting temperature (Tm) values were reported as the peak temperatures of melt endotherms recorded on the second heating scan. Glass transition temperature (Tg) values were recorded as the midpoint of the heat capacity change in the glass transition region during the second heat scan [32,33]. Tm and Tg of CA and PVA were presented in Table 2.

## 3. Results and Discussion

### 3.1. Preparation of CA Microparticles

CA microparticles were prepared by the general procedure represented in Figure 1, where CA/TA binary blend and PVA were intended to be used as dispersed and matrix phases, respectively. The viscosity measurement results for the CA/TA binary system and PVA are illustrated in Figure S2 in Supplement Materials.

The preparation conditions were designed based on Wu’s equation; according to the equation, the particle size of the dispersed phase is mainly governed by three parameters: (i) the interfacial tension (γ_ij_) between the dispersed and matrix phases, (ii) the shear rate (V), and (iii) the viscosity ratio (η_d_/η_m_) between the dispersed and the matrix phases. Since CA/TA/PVA systems were persistently employed in this study, γ_ij_ was not operable for the purpose of manipulating Wu’s equation. Therefore, for the purpose of controlling the particle size, the shear rate and viscosity ratio should be the two major variables. The preparation conditions so designed are summarized in Table 3.

CA microparticles with a particle size of 2–8 μm were successfully obtained after removing the TA and PVA by water. The amount of residual TA and PVA in the CA microparticles obtained by RUN 6 was 21 ppm of TA and 2 ppm, respectively, meaning procedure shown in Figure 1 leads to negligible amounts of TA and PVA in CA microparticles. RUN1–3 are trials investigating the dependence of particle size on shear rate using a dispersed phase composition of CA/TA = 80/20 wt% and PVA_H_ as the matrix phase. Consistent with the suggestions of Wu’s equation, particle sizes decreased with increasing the shear rate. Similarly, RUN6–8 shows the shear rate dependence of particle size with a dispersed phase composition of CA/TA = 83/17 wt% and PVA_L_ as the matrix phase. As expected, particle sizes decreased with increasing the shear rate, in agreement with Wu’s semi-theoretical framework. RUN 2 and RUN 4 were meant to observe the effect of the viscosity ratio on particle size; a smaller viscosity ratio resulted in a smaller particle size; a similar tendency was observed with RUN 3 and RUN 5. While the viscosity ratio shown in Table 3 ranges from 0.62 to 2.87, a ratio outside the range was also tested in preliminary experiments in vain, resulting only in shredded fibers, not particles, suggesting that Wu’s equation served as guidelines for the design of the viscosity ratio. Within the trials shown in Table 3, we could not judge if the unity of the viscosity ratio renders a minimum particle size as implied by Wu’s equation.

**Figure 2:**
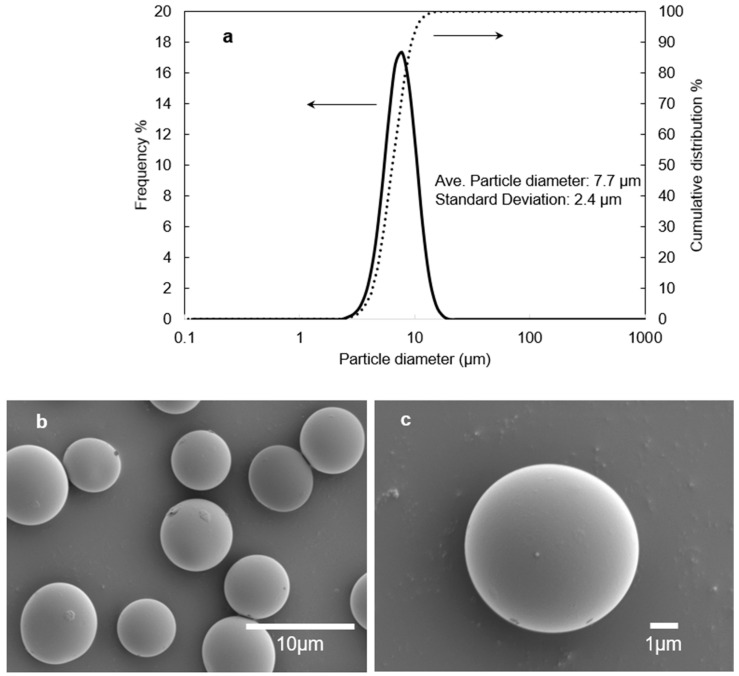
Particles prepared by melt blending at a ratio of 30 wt% of CA/TA = 83/17 and 70 wt% PVA_L_, at a temperature of 230 °C with a rotor speed of 40 rpm (equivalent to a shear rate of 151 s^−1^). (**a**) Number-based particle size distribution of CA; images (**b**,**c**) are scanning electron microscopy images of the particles.

Figure 2 shows particle size distribution and SEM images of CA microparticles obtained by the trial “RUN 6” of Table 3. As shown in Figure 2a, the average particle size was 7.7 ± 2.4 μm (the coefficient of variation of 0.31), which is within the range of 1–15 μm preferred for texture enhancers in cosmetic applications. The SEM observations revealed that the surface of microparticles was smooth without noticeable defects (Figure 2b,c).

### 3.2. Morphology of the Ternary System

Guided by Wu’s equation, we successfully prepared cellulose acetate (CA) microparticles by controlling particle size. Subsequently, a numerical solution of Wu’s equation was obtained and compared with the experimental data. The interfacial tension γ_ij_ used in Wu’s equation was determined using the group contribution method. Specifically, the surface tension components of each substance were calculated based on their molecular structures. From these components, the interfacial tensions between each pair of substances, as well as the expansion coefficients of the two components in the dispersed phase (CA and TA), were obtained. Since the molecular weight dependence of surface tension and its component can be neglected [28], γ, γ^d^, and γ^p^ for PVA_H_ and PVA_L_ are considered equivalent and expressed as PVA. During the calculation of interfacial tension, preliminary experiments were carried out to determine the surface tension and its components at 230 °C or interfacial tension directory between CA/TA and PVA by means of pendant drop method; however, these experiments were unsuccessful due to the high viscosity of the CA/TA blend and PVA [29]. The calculated values are summarized in Table 4.

**Figure 3:**
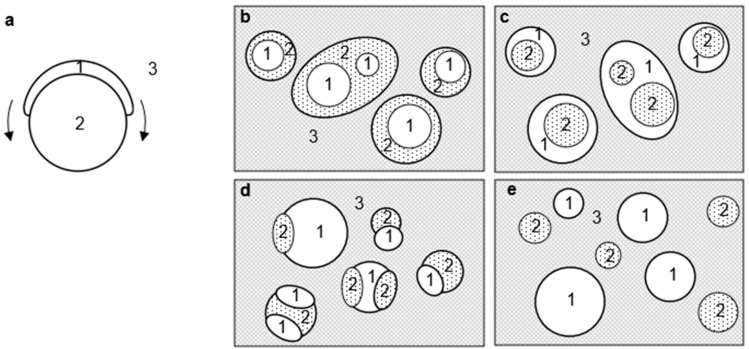
Schematic illustrations (following Nakamura [34]) of morphologies to appear in the melt-blend of materials 1, 2, and 3, depending on spreading coefficients λ_12_ and λ_21_. λ_12_ is the spreading coefficient of dispersed component 1 on dispersed component 2 in matrix 3 (see drawing (**a**)). λ_21_ is the spreading coefficient of dispersed component 2 on dispersed component 1 in matrix 3. (**b**) Encapsulated hybrid particles (1 in 2) corresponding to λ_21_ > 0 following Hobbs [31]. (**c**) Encapsulated hybrid particles (2 in 1) corresponding to λ_12_ > 0. (**d**) Stuck hybrid particles corresponding to λ_21_ < 0 and λ_12_ < 0. (**e**) Isolated particles corresponding to λ_21_ < 0 and λ_12_ < 0.

According to Hobbs, the evaluation of the spreading coefficient enables morphologies shown in Figure 3 [31]. λ_12_ is the spreading coefficient for material 1 on material 2 and describes the physical situation schematically in Figure 3a. in which the ability of material 1 to displace the matrix from the surface of material 2 is considered.

When suffixes are given to CA:1, TA:2, and PVA:3 in Figure 3, the morphologies are evaluated as b (CA is surrounded by TA in PVA matrix), c (TA is surrounded by CA in PVA matrix), d (CA and TA are stacked in PVA matrix), and e (CA and TA are isolated from each other in PVA matrix) [34].

The spreading coefficients of the CA/TA/PVA ternary system were 5.3 and −9.2 for λ_12_ and λ_21_, respectively; the set of positive γ_12_ and negative γ_21_ suggests that CA is capsulated with TA in PVA matrix following Hobbs (Figure 3b). As was suggested with morphology of Figure 3b, γ_23_ was taken as the γ appearing in Wu’s equation.

### 3.3. Comparison Between Particle Sizes Calculated by Wu’s Equation and Observed

**Figure 4:**
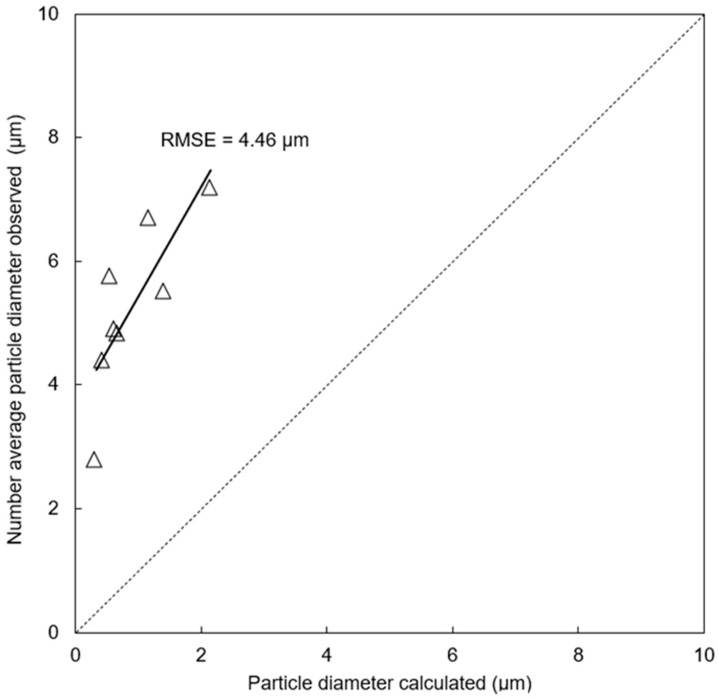
Comparison of the observed and calculated diameter of CA microparticles in this study. The broken line (y = x) represents the ideal 1:1 relationship between calculated and observed values.

In Figure 4, the particle sizes actually observed were compared with numerical solutions by Equation (1).

The root mean squared error (RMSE) between the observed and calculated particle size was 4.46 mm. Considering the target size of 1–15 mm, the prediction accuracy with RMSE of 4.46 mm has room for improvement. Possible reasons for limited prediction accuracy are the following.

- The accuracy of γ calculated by means of group contribution methods has yet to be validated.
- The three interfacial tensions (γ_12_, γ_13_, and γ_23_) for the ternary system may not represent the actual interfacial tension to be incorporated in Wu’s equation.
- Migration of the plasticizer TA from CA dispersed phase to PVA matrix phase may take place affecting the viscosities of both phases changing the viscosity ratio from what was originally designed.

**Figure 5:**
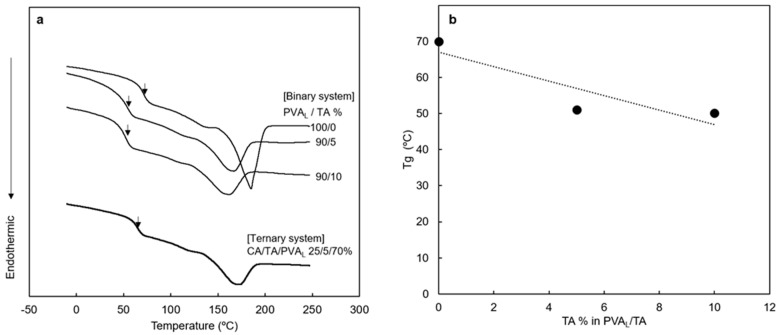
DSC second heating run of the corresponding samples. (**a**) DSC chart with PVA_L_/TA and CA/TA/PVA_L_ = 25/5/70 wt% ternary system. (**b**) Glass transition temperature (Tg) of TA plasticized with PVA_L_.

Validation of Wu’s equation and/or the improvement of prediction accuracy is not altogether within the scope of this study but will require further efforts. Only preliminary observations in relation to the third point (the issue of plasticizer migration) were carried out in this study. For this purpose, differential scanning calorimetry (DCS) studies were carried out for the CA/TA/PVA ternary system and PVA/TA binary systems (Figure 5). It was found with binary systems that the Tg of neat PVA was 71 °C; the Tg decreased with increasing TA (Figure 5b). The Tg corresponding to the PVA phase of the ternary system was 68 °C, which was lower than that of neat PVA, suggesting that the migration of TA took place. A previous study also suggests that triacetin plasticizes polyvinyl alcohol [35].

Since the plot of Tg vs. TA amount was not linear (Figure 5b), further efforts will be required to determine the precise composition of PVA/TA in the ternary system. Such a study will lead to better descriptions of parameters to be incorporated into Wu’s equation facilitating further validation of the equation for melt extrusion of incompatible polymer blends including the ternary system.

## 4. Conclusions

In this study, we prepared cellulose acetate (CA) microparticles through melt extrusion of incompatible polymers blend comprising CA, triacetin (TA), and polyvinyl alcohol (PVA) followed by selective removal of TA and PVA. In so doing, Wu’s equation served as the guidance upon determining the melt extrusion conditions such as shear rate of extrusion and viscosity ratio of dispersed and matrix phases were determined. In our trials, particle size decreased with increasing shear rate and decreasing viscosity ratio, as suggested by Wu’s equation. CA microparticles with a controlled size of 2–8 μm, narrow particle size distribution, and smooth surface were successfully obtained.

The predicted particle sizes were compared with the observed results of our trials: the RMSE was 4.46 μm, which represents a relatively large difference considering that the target particle size range is 1 to 15 μm. Possible reasons for the limited prediction accuracy are (i) lack of accuracy for interfacial tension calculation by group contribution methods and (ii) migration of TA from the dispersed to matrix phase affecting the viscosity ratio. Pending detailed studies, it was found that the TA migration actually takes place.

The CA microparticles with controlled size distribution and smooth surface developed in this study are promising texture enhancers in cosmetic formulations such as liquid foundations and sunscreens.

## 5. Patents

U.S. Patent No. 11,628,134

European Patent No. 3613794

Japanese Patent No. 06609726

Chinese Patent No. ZL201980002492.9

Taiwan Patent No. I719772

Korean Patent No. 10-2111296

“Cellulose acetate particles, cosmetic composition, and method of producing cellulose acetate particles.”

## Figures and Tables

**Table 2:** Materials used in this study.

Material	Role in Ternary System		Properties					
Name	Abbreviation	Role	Suffix Assigned Where Required	Glass Transition Temperature (°C)	Melting Temperature (°C)	Number Average Molecular Weight (Mn) (10^3^ g/mol)	Weight Average Molecular Weight (Mw) (10^3^ g/mol)	Mw/Mn
Cellulose acetate	CA	Dispersed component 1	1	194	227	124	372	3.0
Triacetin	TA	Dispersed component 2 Plasticizer for CA	2	—	—	—	—	—
Polyvinyl alcohol with higher molecular weight	PVA_H_	Matrix ^a^	3	73	188	9.2	22	2.4
Polyvinyl alcohol with lower molecular weight	PVA_L_	Matrix ^a^	3	71	187	6.5	13	20

^a^ Either PVA_H_ or PVA_L_ was employed as matrix in ternary blend of CA/TA/Polyvinyl alcohol.

**Table 3:** Experimental conditions and results for the preparation of CA microparticles.

ID	Dispersed Phase Component CA/TA Ratio wt%	Matrix Phase Component PVA Type -	Dispersed/Matrix Ratio wt%	Viscosity Ratio	Shear Rate (S^−1^)	Particle Size
				-		(μm)
RUN 1	80/20	PVA_H_	30/70	0.75	151	4.9
RUN 2	80/20	PVA_H_	30/70	0.72	303	4.4
RUN 3	80/20	PVA_H_	30/70	0.62	606	2.8
RUN 4	80/20	PVA_L_	30/70	1.67	303	6.7
RUN 5	80/20	PVA_L_	30/70	1.29	606	5.8
RUN 6	83/17	PVA_L_	30/70	2.87	151	7.7
RUN 7	83/17	PVA_L_	30/70	2.09	303	5.5
RUN 8	83/17	PVA_L_	30/70	1.51	606	4.8

**Table 4:** Surface tension, interfacial tension and spreading coefficient at 230 °C for CA, TA, and PVA.

	Material	CA	TA	PVA
	ID	1	2	3
Surface tension and its components (mJ/m^2^)	γ	18.8	11.4	36.5
	γ^d^	9.4	6.4	8.6
	γ^p^	9.5	4.9	27.9
Interfacial tension (mJ/m^2^)	γ_12_	2.0		N.A.
	γ_13_	9.1	N.A.	9.1
	γ_23_	N.A.	16.4	
Spreading coefficient (mJ/m^2^)	λ_12_	5.3		
	λ_21_	−9.2		

Spreading coefficient λ_ij_: spreading coefficient of j on i in matrix other than i or j. N.A.: not applicable.

## Data Availability

The authors declare that the data supporting the findings of this study are available within the paper and its Supplementary Information files.

## Supplementary Materials

The following supporting information can be downloaded at: Available online: https://www.mdpi.com/article/10.3390/polym17152118/s1, Table S1. Solubility parameter component group contribution (Van Krevelen method) [27]; Table S2. Parachor group contribution (Quayle value) [25]; Table S3. Van der Waals volume [25]; Table S4. Number of groups i in the repeat unit and calculated values for each component to estimate surface tension at 230 °C; Figure S1. Viscosity vs. shear rate at 230 °C. CA plasticized with 17 wt% of TA (filled circles), CA plasticized with 20 wt% of TA (open circles), PVAH (filled triangles), and PVAL (filled triangles). Compositions of ternary systems are expressed on a weight basis; Figure S2. Algorithm for calculations of surface tension components, interfacial tension γij, and spreading coefficient λij following (a) van Krevelen, (b) Quayle, (c) Lee, (d) Wu, and (e) Hobbs at blending temperature of 230 °C. References [26,36] are cited in the Supplementary Materials.
