# Supplementary material for: Development of Cellulose Acetate Spherical Microparticles by Means of Melt Extrusion of Incompatible Polymer Blend
Source: Polymers (Basel). 2025 Jul 31;17(15):2118. doi: 10.3390/polym17152118 (PMC12349337; doi:10.3390/polym17152118)
Supplement: Supplementary file 1 [file polymers-17-02118-s001.zip › polymers-3744371-supplementary.pdf]

Supplementary Material

Journal: ***Polymers***

Development of Cellulose Acetate Spherical Microparticles by means of Melt Extrusion of Incompatible Polymer Blend

Masaya Omura[1,2,*], Keiko Kobayashi[3], Kanji Nagai[2,4], Shu Shimamoto[2,5]

[1] *Business Strategy, Healthcare SBU, Daicel Corporation, Minato-ku, Tokyo 108-0075, Japan*

[2]*Graduate School of Natural Science and Technology, Kanazawa University, Kanazawa-Shi, Ishikawa 920-1192, Japan*

[3]*CAFBLO Business Strategy, Material SBU, Daicel Corporation, Himeji-Shi, Hyogo 671-1283, Japan*

[4]*Life Sciences R&D Center, PharmaTek BU, Life Sciences SBU, Daicel Corporation, Myoko-Shi, Niigata 944-8550, Japan*

[5]*Business Development Center, Innovation and Business Development Headquarters, Daicel Corporation, Minato-ku, Tokyo 108-0075, Japan*

[*]**Corresponding author:** Masaya Omura; *Business Strategy, Healthcare SBU, Daicel Corporation, Minato-ku, Tokyo 108-0075, Japan; Graduate School of Natural Science and Technology, Kanazawa University, Kanazawa-Shi, Ishikawa 920-1192, Japan*; Tel.: +81-8024862112; Fax: +81-792744086; E-mail: m-omura@jp.daicel.com.

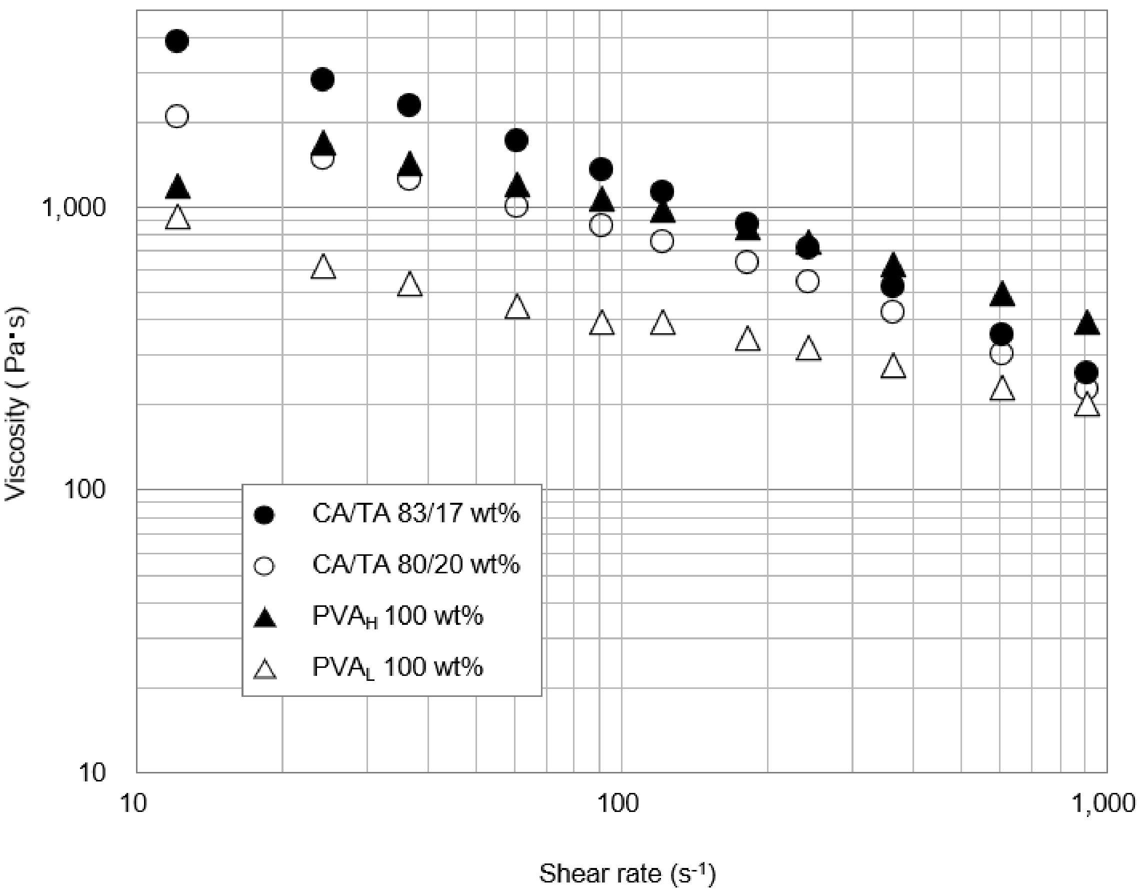

Figure S1. Viscosity vs. shear rate at 230ºC. CA plasticized with 17 wt% of TA (filled circles), CA plasticized with 20 wt% of TA (open circles), PVA_H (filled triangles), and PVA_L (filled triangles). Compositions of ternary systems are expressed on weight basis.

Table S1. Solubility parameter component group contribution (Van Krevelen method). (Van Krevelen and Nijenhuis 2009b)

	F_{di} $(MJ/m^3)^{1/2}/mol$	F_{pi} $(MJ/m^3)^{1/2}/mol$	E_{hi} J/mol
$-CH_3$	420	0	0
$-CH_2-$	270	0	0
>CH-	80	0	0
-OH	210	770	20000
-O-	100	400	3000
-COO-	390	490	4500
Ring	190	0	0

F represents molar attraction constant, and E represents hydrogen bonding energy for the group contribution method.

d, p, and h are the disperse component, polar component, and hydrogen bonding component, respectively.

Table S2. Parachor group contribution (Quayle value) (Quayle 1953).

	Parachor P_i $(MJ/m^3)^{1/2}/mol$
$-CH_3$	55.5
$-CH_2-$	40.0
>CH-	24.5
-OH	32.8
-O-	17.3
-COO-	54.8
Ring	-

Table S3. Van der Waals volume (Van Krevelen and Nijenhuis 2009a)

	Van der Waals volume V_{wi} cm^3/mol
$-CH_3$	13.7
$-CH_2-$	10.2
>CH-	6.8
-OH	8.0
-O-	5.5
-COO-	15.2

Ring	-

Table S4. Number of group *i* in the repeat unit and calculated values for each component to estimate surface tension at 230 °C.

		CA	TA	PVA	Formula used for calculation
Number of group i presented repeat unit	$-CH_3$	2.45	3.00	0.01	
	$-CH_2-$	1.00	2.00	1.05	
	>CH-	5.00	1.00	1.05	
	-OH	0.55	-	1.04	
	-O-	2.00	-	-	
	-COO-	2.45	3.00	0.01	
	Ring	1.00	-	-	
Molar volume below T_g	V_g cm³/mol	208.6	182.2	42.2	$V_g = 1.6 \times V_w$ (Van Krevelen and Nijenhuis 2009a)
Thermal expansion ratio	E_g	0.059	-	0.012	$E_g = 0.45 \times 10^{-3} V_w$ (Van Krevelen and Nijenhuis 2009a)
	E_l	0.130	-	0.026	$E_l = 1.00 \times 10^{-3} V_w$ (Van Krevelen and Nijenhuis 2009a)
Molar volume at 230 ℃	V cm³/mol	222.3	237.1	47.0	$V_{CA, PVA} = V_g + E_g(Tg - 25) + E_l(T_{230} - Tg)$ (Van Krevelen and Nijenhuis 2009a)<br>$V_{TA} = M / (-0.0011xT_{230} + 1.1801)$ (Deng et al. 2018)
Estimated solubility parameter and each component at 230 ℃	δ_d $(MJ/m^3)^{1/2}$	13.6	12.87	14.17	
	δ_p $(MJ/m^3)^{1/2}$	6.30	6.20	12.46	
	δ_h $(MJ/m^3)^{1/2}$	12.10	9.41	22.35	
	$\delta (MJ/m^3)^{1/2}$	19.24	17.10	29.26	
Estimated surface tension and each component at 230℃	γ^d mJ/m²	9.35	6.43	8.57	$1 - P = \gamma^d/\gamma = (\delta_d/\delta)^{1/0.54}$ (Lee 1970, Van Krevelen and Nijenhuis 2009c)
	γ^p mJ/m²	9.47	4.94	27.94	
	γ mJ/m²	18.83	11.37	36.51	$\gamma = (Ps / V)^4$ (Quayle 1953)

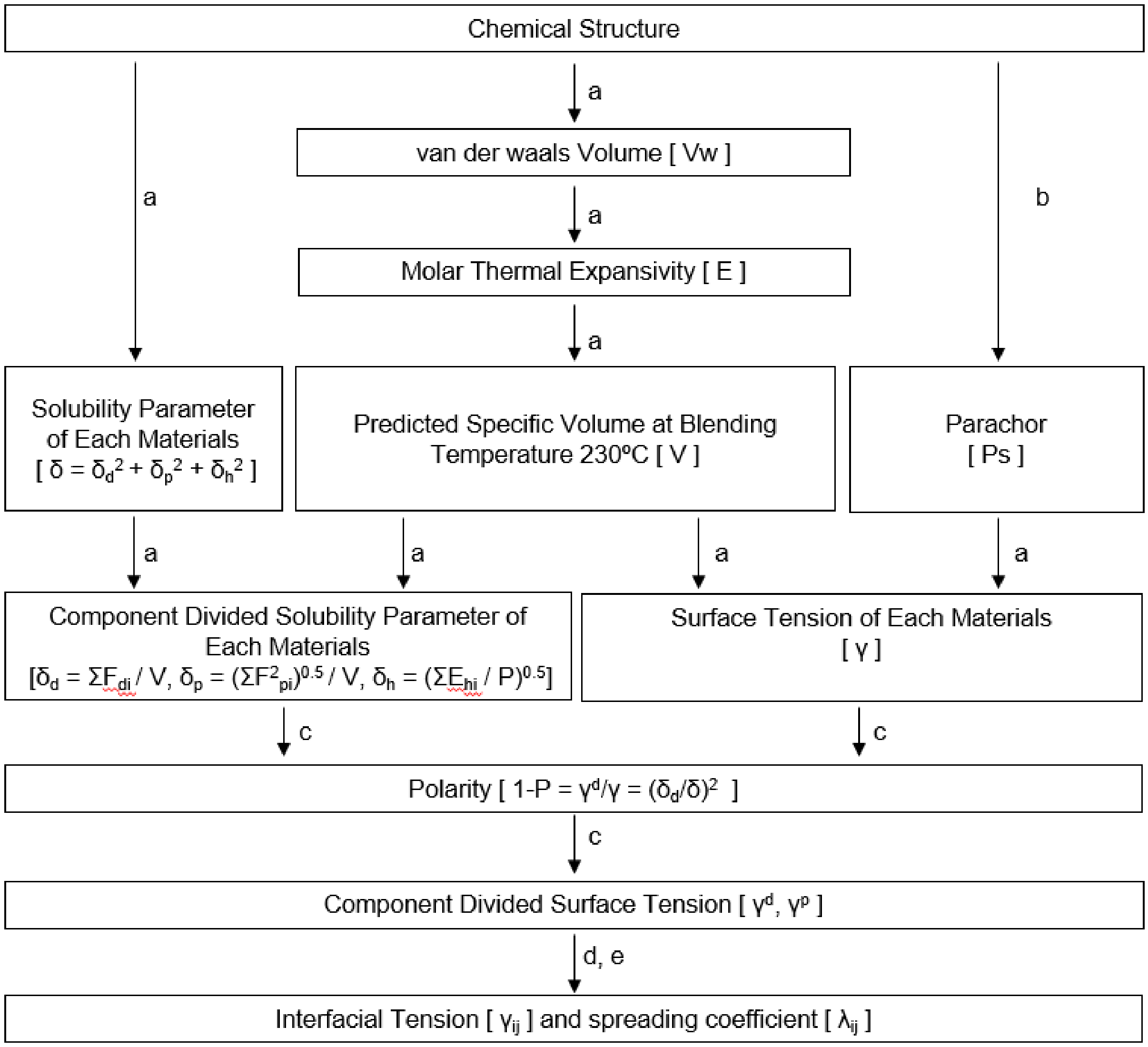

Figure S2. Algorithm for calculations of surface tension components, interfacial tension γij, and spreading coefficient λij after a) van Krevelen, b) Quayle, c) Lee, d) Wu and e) Hobbs at blending temperature (230ºC)

References

Deng D, Liu X, Cui Y, Jiang Y (2018) Investigation of SO_2 solubilities in some biobased solvents and their thermodynamic properties. J Chem Thermodyn 119:84–91. https://doi.org/10.1016/j.jct.2017.12.021

Lee LH (1970) Relationships between solubility parameters and surface tensions of liquids. J Paint Technol 42:365–370.

Quayle OR (1953) The parachors of organic compounds. An interpretation and catalogue. Chem Rev 53:439–589. https://doi.org/10.1021/cr60166a003

Van Krevelen DW, Nijenhuis KT (2009a) Chapter 4 - Volumetric Properties. In: Properties of Polymers, Fourth Edition. Elsevier, Amsterdam, pp 71–108. https://doi.org/10.1016/B978-0-08-054819-7.00004-2

Van Krevelen DW, Nijenhuis KT (2009b) Chapter 7 - Cohesive Properties and Solubility. In: Properties of Polymers, Fourth Edition. Elsevier, Amsterdam, pp 189–227. https://doi.org/10.1016/B978-0-08-054819-7.00007-8

Van Krevelen DW, Nijenhuis KT (2009c) Chapter 8 - Interfacial Energy Properties. In: Properties of Polymers, Fourth Edition. Elsevier, Amsterdam, pp 229–244. https://doi.org/10.1016/B978-0-08-054819-7.00008-X
